# Conditional essentiality of the 11-subunit complex I-like enzyme in strict anaerobes: the case of *Desulfitobacterium hafniense* strain DCB-2

**DOI:** 10.3389/fmicb.2024.1388961

**Published:** 2024-06-26

**Authors:** Mathilde Stéphanie Willemin, Florence Armand, Romain Hamelin, Julien Maillard, Christof Holliger

**Affiliations:** ^1^Laboratory for Environmental Biotechnology (LBE), Ecole Polytechnique Fédérale de Lausanne (EPFL), Lausanne, Switzerland; ^2^Proteomic Core Facility (PCF), Ecole Polytechnique Fédérale de Lausanne (EPFL), Lausanne, Switzerland

**Keywords:** complex I-like enzyme, *Desulfitobacterium*, energy metabolism, bacterial respiration, rotenone, respiratory complex I, anaerobic respiratory processes

## Abstract

In oxidative phosphorylation, respiratory complex I serves as an entry point in the electron transport chain for electrons generated in catabolic processes in the form of NADH. An ancestral version of the complex, lacking the NADH-oxidising module, is encoded in a significant number of bacterial genomes. Amongst them is *Desulfitobacterium hafniense*, a strict anaerobe capable of conserving energy via organohalide respiration. This study investigates the role of the complex I-like enzyme in *D. hafniense* energy metabolism using rotenone as a specific complex I inhibitor under different growth conditions. The investigation revealed that the complex I-like enzyme was essential for growth with lactate and pyruvate but not in conditions involving H_2_ as an electron donor. In addition, a previously published proteomic dataset of strain DCB-2 was analysed to reveal the predominance of the complex under different growth conditions and to identify potential redox partners. This approach revealed seven candidates with expression patterns similar to Nuo homologues, suggesting the use of diverse electron sources. Based on these results, we propose a model where the complex I-like enzyme serves as an electron entry point into the respiratory chain for substrates delivering electrons within the cytoplasm, such as lactate or pyruvate, with ferredoxins shuttling electrons to the complex.

## Introduction

Respiratory complex I, or NADH:ubiquinone oxidoreductase, is well known for its central role in aerobic respiration as it generates a proton motive force by coupling the transfer of two electrons from NADH to the quinone pool whilst pumping four protons across the cytoplasmic membrane (Hirst, 2013; Friedrich et al., 2016). Although the mitochondrial enzyme consists of a total of 44 subunits, it shares 14 core subunits (NuoA-N, *Escherichia coli* nomenclature) with the bacterial version of the complex (Baradaran et al., 2013; Vinothkumar et al., 2014). The enzyme is generally divided into three functional modules: the NADH-oxidising (N) module, the quinone-reducing (Q) module, and the proton-pumping (P) module. The N-module is situated at the tip of the peripheral arm and consists of the NuoEFG subunits. It comprises the NADH-binding site, where electrons are delivered to a non-covalently bound flavin mononucleotide (FMN) cofactor held in NuoF (Brandt, 2006; Friedrich, 2014; Gnandt et al., 2016). From there, the electrons are tunnelled through a chain of seven FeS clusters to the quinone-reducing site (Q-module) at the membrane side of the peripheral arm (Hayashi and Stuchebrukhov, 2011). The quinone-binding site, formed by NuoABCDH, comprises the last two FeS centres that transfer the electron to the quinone molecule (Baradaran et al., 2013; Friedrich, 2014) and is the target of complex I inhibitors (Singer, 1979; Hu et al., 2013; Degli Esposti, 2016). Finally, the P-module, which does not harbour any redox cofactor, is dedicated to proton translocation (Baradaran et al., 2013).

The consensus evolutionary model of complex I suggests an ancestor, common to both complex I and membrane-bound hydrogenases, that is composed of 11 subunits and is likely evolved to become the last common ancestor of the 14-subunit complex I family (Moparthi and Hägerhäll, 2011; Brandt, 2019; Parey et al., 2020). The 11-subunit enzyme, which is initially described in photosynthetic systems such as chloroplasts and cyanobacteria (Friedrich and Scheide, 2000), was shown to be more widespread than initially thought and was present in the genome of members of diverse eubacterial phyla, including the Firmicutes (Moparthi and Hägerhäll, 2011; Degli Esposti, 2016). This version of the complex lacks the N-module (i.e., NuoEFG), but clearly differentiates from the membrane-bound hydrogenases. Sequence alignments suggested that the Q-module has the same function in the 11- and 14-subunit versions, which led to the proposition that the 11-subunit complex I-like enzyme is the actual energy-coupling engine that may have acquired the N-module only later in evolution (Moparthi and Hägerhäll, 2011). The N-module is considered by Moparthi et al. as an electron donor moiety, the acquisition of which allowed the respiratory chain to receive electrons from NADH. The question of the direct electron donor that is involved in the 11-subunit complex I-like enzyme remains. An attempt to find a common electron-donating protein that would be conserved in all bacteria harbouring the 11-subunit complex was not successful. Furthermore, it was hypothesised that the 11-subunit version of the complex may work with several partners, acting as a docking platform for various electron donors (Moparthi and Hägerhäll, 2011).

**Figure 1:**
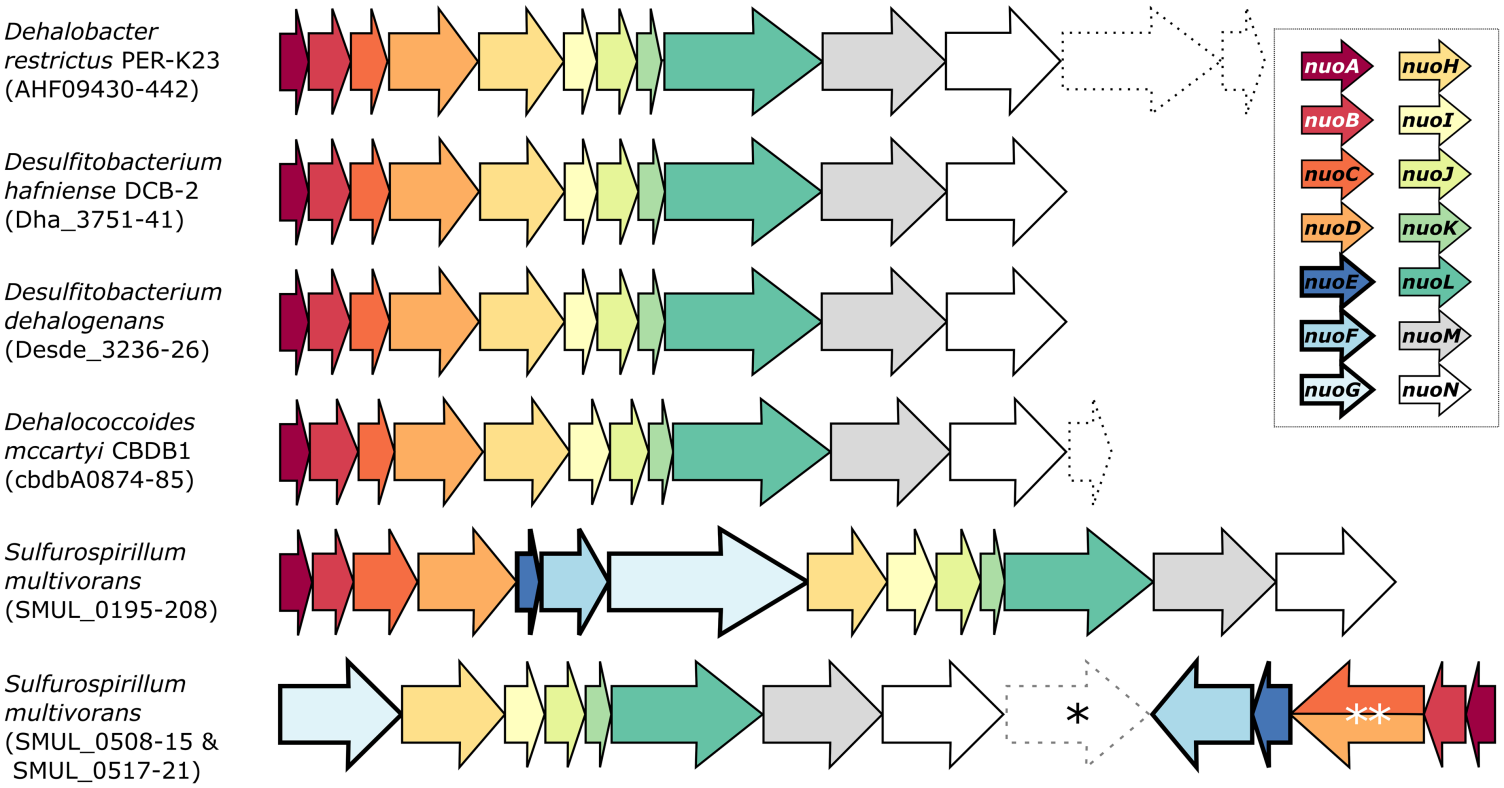
Genetic organisation of the complex I and complex I-like operons in selected organohalide-respiring bacteria. The nomenclature of the NADH:ubiquinone oxidoreductase (*nuo*) subunits was used here. Dashed arrows indicate the presence of additional genes of various functions within the *nuo* transcriptional unit. Note that SMUL_0516 (indicated by an asterisk) is a gene of unknown function that is not predicted to be part of any *S. multivorans* nuo operons. The double asterisk indicates a *nuoCD* fused gene (SMUL_0519). Genomic predictions were taken from biocyc.org.

*Desulfitobacterium* spp. are versatile anaerobic bacteria from the Firmicutes phylum [recently renamed as Bacillota (Oren and Garrity, 2021)] and are capable of using various molecules as electron donors and acceptors, including several organohalogens, thus making *Desulfitobacterium* spp. important bacteria for bioremediation. Their genome encodes for an 11-subunit complex I-like enzyme (Nonaka et al., 2006; Kim et al., 2012; Kruse et al., 2015). For example, the genome of *D. hafniense* strain DCB-2 harbours a *nuoABCDHIJKLMN* operon (with the locus ACL21757-67) that clearly lacks *nuoE*, *F,* and *G* (Figure 1).

Whilst a separate three-gene operon (ACL19452-4) was initially annotated as *nuoEFG* in the genome of strain DCB-2, it rather shows similarity to a cytoplasmic NAD^+^-dependent formate dehydrogenase (FDH) such as the FdsABG complex from *Cupriavidus necator* (Young et al., 2020). Sequence alignments of ACL19452-4 with the selected members of homologous proteins are depicted in Supplementary Figure S1. Briefly, ACL19454, which is the FDH catalytic unit, shares features of FdsA from *C. necator*, FdhA from *Moorella thermoacetica,* and FdhF from *E. coli* (Supplementary Figure S1A). In contrast, the alignment of ACL19454 with NuoG representatives shows that only the FeS cluster motifs in the N-terminal region are conserved (Supplementary Figure S1B), clearly excluding ACL19454 from taking part in complex I. A sequence alignment of ACL19453 with a selection of homologous proteins (including *E. coli* NuoF) further indicates its similarity to the β-subunit of FDH, whilst NuoF lacks the N-terminal motif for a 2Fe-2S cluster and the C-terminal motif for a 4Fe-4S cluster (Supplementary Figure S1C). The alignment of ACL19452 with sequences of the FDH ferredoxin subunit and NuoE, however, does not distinguish between FDH and complex I (Supplementary Figure S1D). Finally, the structural alignment of ACL19452-3 from *D. hafniense* strain DCB-2 with *C. necator* FdsBG largely confirms its homology to FDH enzymes (Supplementary Figure S1E). Therefore, ACL19452, ACL19453, and ACL19454 can be reannotated as the subunits of FDH and are likely not involved in electron transfer to the complex I-like enzyme in *D. hafniense*. A similar 11-subunit complex I-like enzyme was also found in other organohalide-respiring bacteria (OHRB), such as members of the genera *Dehalobacter* and *Dehalococcoides* (Figure 1) (Kube et al., 2005; Seshadri et al., 2005; Kruse et al., 2013). Moreover, proteomic analyses have identified multiple subunits of the complex I-like enzymes, such as in *Dehalococcoides mccartyi* strain CBDB1 (subunits NuoBCDHIMN) when cultivated on hexachlorobenzene (Schiffmann et al., 2014), and in *Dehalobacter restrictus* strain PER-K23 (subunits NuoBCD) when growing with tetrachloroethene (PCE) as terminal electron acceptor (Rupakula et al., 2013). On the other hand, and as shown in Figure 1, *Sulfurospirillum multivorans* harbours two different versions of complex I (Goris et al., 2015). The first one is a classical 14-subunit complex I, whilst the second one is an atypical version usually found in ε-proteobacteria. The latter is encoded in two operons (*nuoGHIJKLMN* and *nuoABCDEF*) in opposite directions in the genome and is likely receiving electrons from ferredoxin or flavodoxin (Weerakoon and Olson, 2008). Proteomic analysis of *S. multivorans* has revealed that both versions of the complex are expressed in cells cultivated under various respiratory conditions, including OHR (Goris et al., 2015).

The role of the 11-subunit complex I-like enzyme in OHR and other anaerobic respiratory processes has not yet been specifically investigated to date. In the present study, we addressed this question using two different approaches. On the one hand, rotenone was used as a typical complex I inhibitor to evaluate the importance of the complex I-like enzyme for the growth of *Desulfitobacterium hafniense* strain DCB-2. Several growth conditions were tested in parallel in order to measure the effect of rotenone on cells using different energy metabolisms, including OHR. On the other hand, the comparative proteomic dataset of *Desulfitobacterium hafniense* strain DCB-2 that we have published recently (Willemin et al., 2023) was used to compare the relative abundance of the complex I-like enzyme under different growth conditions and to identify proteins possibly involved in donating electrons to this complex.

## Materials and methods

### Bacterial cultivation

*Desulfitobacterium hafniense* strain DCB-2 was cultivated in 40 mL or 200 mL anaerobic flasks following the media preparation as detailed previously by Willemin et al. (2023). In short, for the six growth conditions tested, the same basal medium was used. Prior to inoculation, electron donors and acceptors were added to the latter in order to trigger different metabolic pathways. Pyruvate was used for fermentative metabolism at a final concentration of 40 mM (Py-only), while combinations of 20 mM of pyruvate or lactate with 20 mM of fumarate stimulated respiratory metabolism (Py/Fu or La/Fu, respectively). For triggering the OHR metabolism, 10 mM of 3-chloro-4-hydroxyphenylacetic acid (ClOHPA) served as the electron acceptor in combination with 20 mM of lactate (La/ClOHPA). Alternatively, molecular hydrogen was added in the gas phase to serve as an electron donor, either with fumarate (H_2_/Fu) or ClOHPA (H_2_/ClOHPA) as an electron acceptor and with the addition of 2 mM acetic acid as a carbon source. Small variations from the basal recipe were made to adapt the growth medium to *D. hafniense* strain TCE1 (DSM12704) or *Dehalobacter restrictus* strain PER-K23 (DSM 9455). In those cases, 0.1 g/L of peptone was used instead of yeast extract in the basal solution. In addition, tetrachloroethene (PCE) was used as an electron acceptor for the latter strains, but each one of them required a different preparation. PCE stock solutions were prepared in hexadecane and added after inoculation on top of the aqueous phase, resulting in a bi-phasic system to sustain a constant and slow diffusion of PCE to the aqueous phase and avoid any toxic effect on the growth of PCE in the direct environment of cells. For *D. restrictus*, 4% (v/v) of 500 mM PCE in hexadecane was added to the culture flask, whilst 1% (v/v) of 2 M was used for *D. hafniense* strain TCE1, resulting in a nominal concentration of 20 mM both cases. H_2_ was used as the electron donor for both strains (H_2_/PCE). When applicable, 20 mM lactate was alternatively used as the electron donor for strain TCE1 cultures (La/PCE).

Additionally, the cultures were treated with 10 μM of rotenone or piericidin A (a stock solution of 10 mM prepared in ethanol). The concentration was chosen based on existing literature (Meijer et al., 1978; Darrouzet and Dupuis, 1997; Scheide et al., 2002; Kang et al., 2016). In the case of spike experiments, rotenone was spiked in the culture flasks after the start of the exponential growth phase to clearly capture the phenotype of growth inhibition but early enough to avoid confusion with the entry into the stationary phase. Ethanol was either added or spiked in the control cultures. Growth was followed spectrophotometrically by measuring the absorbance of culture samples at 600 nm (OD_600_).

### The analysis of the proteomic dataset

The dataset used to identify possible protein partners to the complex I-like enzyme in *D. hafniense* strain DCB-2 was published earlier (Willemin et al., 2023). The dataset is available in the ProteomeXchange Consortium (www.ebi.ac.uk/pride; accession number: PXD030393). The Z-scores (or standard scores) of each detected protein were calculated across the six growth conditions with the formula: Z-score = (x – μ)/σ, where x corresponds to the relative abundance of the protein of interest in a given sample; μ represents the mean of abundance values across all 18 samples (i.e., 6 conditions in triplicate); and σ represents the standard deviation of abundances. The median of the Z-scores from the 10 Nuo homologous subunits detected in the proteomic dataset was calculated and was used to identify potential complex I-like partner proteins in the dataset. The Euclidean distance to the median was calculated for all the proteins in the dataset using the Euclidean method of the distance matrix computation (dist) function from the stats package in R. This method enables the calculation of the distance between two vectors following this formula: $\mathrm{dist}=\sqrt{\underset{i}{\sum }{\left(x_{i}-y_{i}\right)}^{2}}\text{,}$ where x is the median Z-score of the Nuo homologues in the 18 biological samples and y is the Z-score of a given protein to be tested.

Proteins were ranked by their Euclidean distance to the median. To evaluate the possible involvement of these proteins in electron transfer to the complex I-like enzyme, their predicted sequence and function were analysed for redox activity using BlastP (Altschul et al., 1990), InterPro (Paysan-Lafosse et al., 2023), and MetalPredator (Valasatava et al., 2016).

## Results

### The inhibition of the 11-subunit complex I-like enzyme results in growth defects in specific conditions

**Figure 2:**
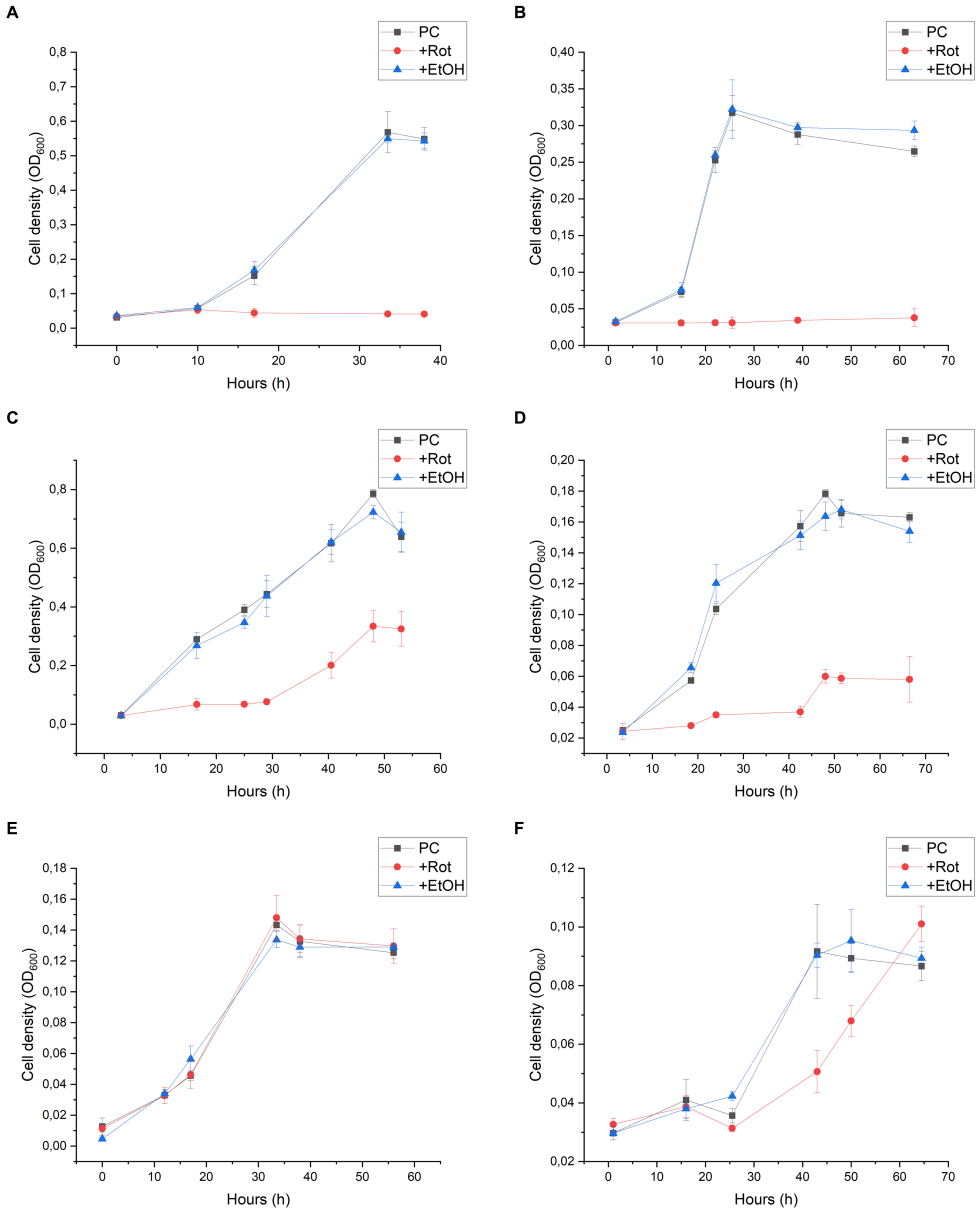
Rotenone inhibition on the growth of *Desulfitobacterium hafniense* strain DCB-2 in the following conditions: **(A)** pyruvate/fumarate; **(B)** lactate/fumarate; **(C)** pyruvate-only; **(D)** lactate/ClOHPA; **(E)** hydrogen/fumarate; and **(F)** hydrogen/ClOHPA. For each condition, triplicates of three different cultures were performed: positive control (PC), with the supplementation of rotenone dissolved in ethanol (+Rot), and with ethanol only (+EtOH). Growth was monitored by measuring cell density at 600 nm (OD_600_) over time.

Rotenone is considered to be a strong inhibitor of complex I of the respiratory chain in mitochondria and bacteria. The mechanism of action comprises the inhibition of electron transfer from the iron–sulfur centres in complex I to ubiquinone and menaquinone. As this inhibition is not dependent on the NADH oxidising module, we used this inhibitor to get indications on the role of the 11-subunit complex I-like enzyme lacking the N-module in the energy metabolism of *D. hafniense* strain DCB-2 (Schiller and Zickermann, 2022). The effect of rotenone was measured by the growth of *D. hafniense* strain DCB-2 cultivated in six different conditions. The conditions were chosen in order to trigger different metabolisms: fermentative conditions with pyruvate (Py-only), fumarate respiration using pyruvate (Py/Fu), lactate (La/Fu) or hydrogen (H_2_/Fu) as electron donors, and organohalide respiration using ClOHPA as electron acceptor with lactate or hydrogen as electron donor (La/ClOHPA and H_2_/ClOHPA, respectively). Rotenone dissolved in ethanol was added to the cultures at the time of inoculation, and the growth was monitored over time (Figure 2, +Rot). Control experiments were performed by adding the same volume of ethanol only (+EtOH) or without any amendment (positive control, PC). All growth experiments were performed in triplicates.

Strain DCB-2 showed different behaviours in response to rotenone depending on the growth conditions. The conditions with organic electron donors (pyruvate or lactate) displayed a clear growth defect (Figures 2A–D). When rotenone was applied to Py/Fu and La/Fu conditions (panels A and B, respectively), the growth was completely stopped, whilst the two corresponding controls reached a maximum OD_600_ value of approximately 0.6 and 0.3, respectively. In contrast, the Py-only culture (panel C) treated with rotenone showed an initial lag phase of approximately 30 h, but then recovered to half the level of the controls with a similar growth rate. In the La/ClOHPA condition (panel D), the growth was also initially inhibited but the strain grew to a smaller extent after 20 h, reaching a cell density corresponding to 30% (OD_600_ = 0.055) of the density observed in the control batch cultures (OD_600_ = 0.170). Interestingly, the two growth conditions using H_2_ as an electron donor were not or only slightly affected by the addition of rotenone (Figures 2E,F). No growth defect was observed for strain DCB-2 growing with H_2_ and fumarate (panel E), whilst only a small decrease in the initial growth rate was observed for H_2_/ClOHPA as compared to the controls, but without any consequence on the final cell density reached (panel F). In all the above-mentioned experiments, the ethanol-treated cultures did not show any growth difference in comparison to the untreated positive controls. Therefore, the ethanol treatment was used as the only control for further experiments. The different growth phenotypes above for rotenone were confirmed by performing the same experiments in Py-only, La/Fu, and H_2_/Fu conditions but using piericidin A instead, which is another complex I inhibitor targeting the quinone-binding site (Schiller and Zickermann, 2022). The growth of strain DCB-2 remained unaffected in the H_2_/Fu condition but was drastically reduced in the La/Fu condition and less but significantly affected in the Py-only condition (Supplementary Figure S2). A similar experiment was performed with two additional OHRB strains from the Bacillota phylum (previously Firmicutes) for which a proteomic dataset that indicated the expression of their complex I-like enzyme was available. First, the obligate OHRB *Dehalobacter restrictus* strain PER-K23 is restricted to H_2_ and PCE as an electron donor and acceptor, respectively (Rupakula et al., 2013). Second, *Desulfitobacterium hafniense* strain TCE1 shows a versatile energy metabolism and is cultivated with the same electron acceptor as *D. restrictus* but with either lactate or hydrogen as an electron donor (unpublished dataset, G. Buttet and J. Maillard). As expected, *D. restrictus* PER-K23 did not show any rotenone-induced growth defect (Supplementary Figure S3). In contrast, for *D. hafniense* TCE1 (Supplementary Figure S4), rotenone induced a clear growth inhibition in cells cultivated with lactate (panel A), whilst H_2_ allowed the cells to grow in the presence of rotenone, however, with a lower growth rate and extent in comparison to the control experiment (panel B).

**Figure 3:**
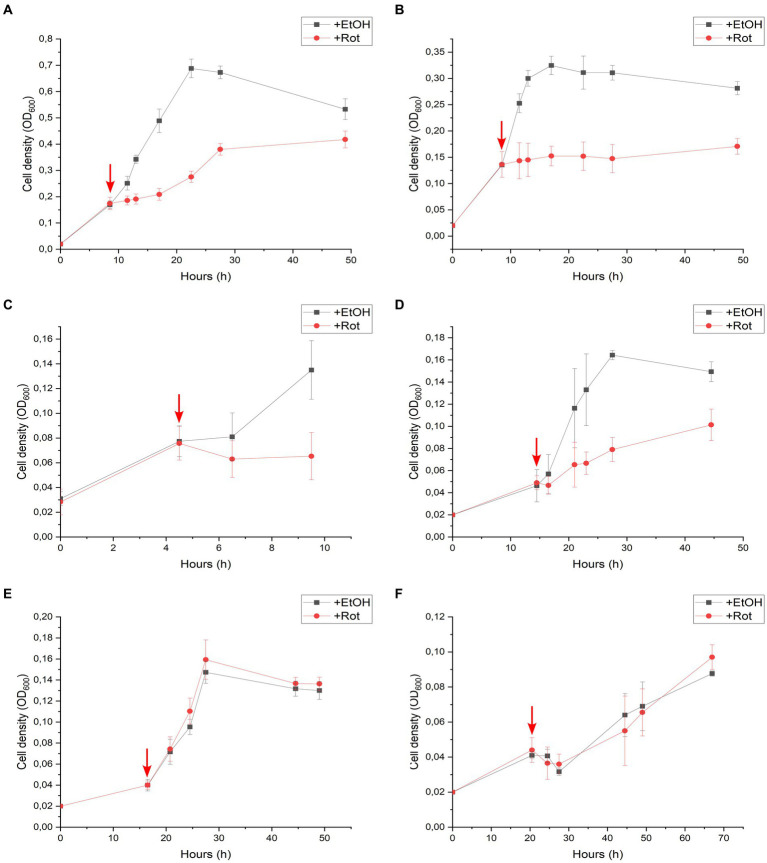
Rotenone spike experiments of *Desulfitobacterium hafniense* strain DCB-2 cultivated in the following conditions: **(A)** pyruvate/fumarate; **(B)** lactate/fumarate; **(C)** pyruvate-only; **(D)** lactate/ClOHPA; **(E)** hydrogen/fumarate; and **(F)** hydrogen/ClOHPA. For each condition, triplicates of two different cultures were spiked with ethanol as control (+EtOH), or with rotenone dissolved in ethanol (+Rot). The red arrows indicate the time point at which the cultures were spiked. Growth was monitored by measuring cell density at 600 nm (OD_600_) over time.

An additional series of batch cultures was performed with strain DCB-2, in which rotenone was spiked in the cultures after growth had started. The same six growth conditions as above were monitored, and the results are displayed in Figure 3. In the absence of rotenone and depending on the growth conditions, strain DCB-2 neither grows at the same rate nor to the same extent. Therefore, the time for spiking rotenone in the cultures was adapted to each condition, as indicated by a red arrow in the panels of Figure 3. The results obtained here reflected the ones obtained during the first experimental approach. After the spike of rotenone, the cultures growing in Py/Fu, La/Fu, Py-only, and La/ClOHPA conditions either completely stopped growing or showed a strong reduction in the growth rate and extent in comparison to the positive controls (Figures 3A–D, respectively). Furthermore, the growth of the cultures in H_2_/Fu and H_2_/ClOHPA was not affected (Figures 3E,F).

The growth of strain DCB-2 in La/Fu condition was further investigated as it showed one of the strongest phenotypes in response to rotenone (Figure 2B). First, rotenone and hydrogen were supplemented in the culture before inoculation (Supplementary Figure S5A), and in the second, rotenone was first spiked in the growing cultures, while hydrogen was spiked after the growth of treated cultures started to decline (Supplementary Figure S5B). In both experiments, H_2_ addition, although only partially, helped strain DCB-2 to grow in the presence of rotenone, suggesting that the bacteria switched metabolism to use H_2_ instead of lactate as the electron donor.

### The identification of potential complex I-like enzyme protein partners in strain DCB-2

**Figure 4:**
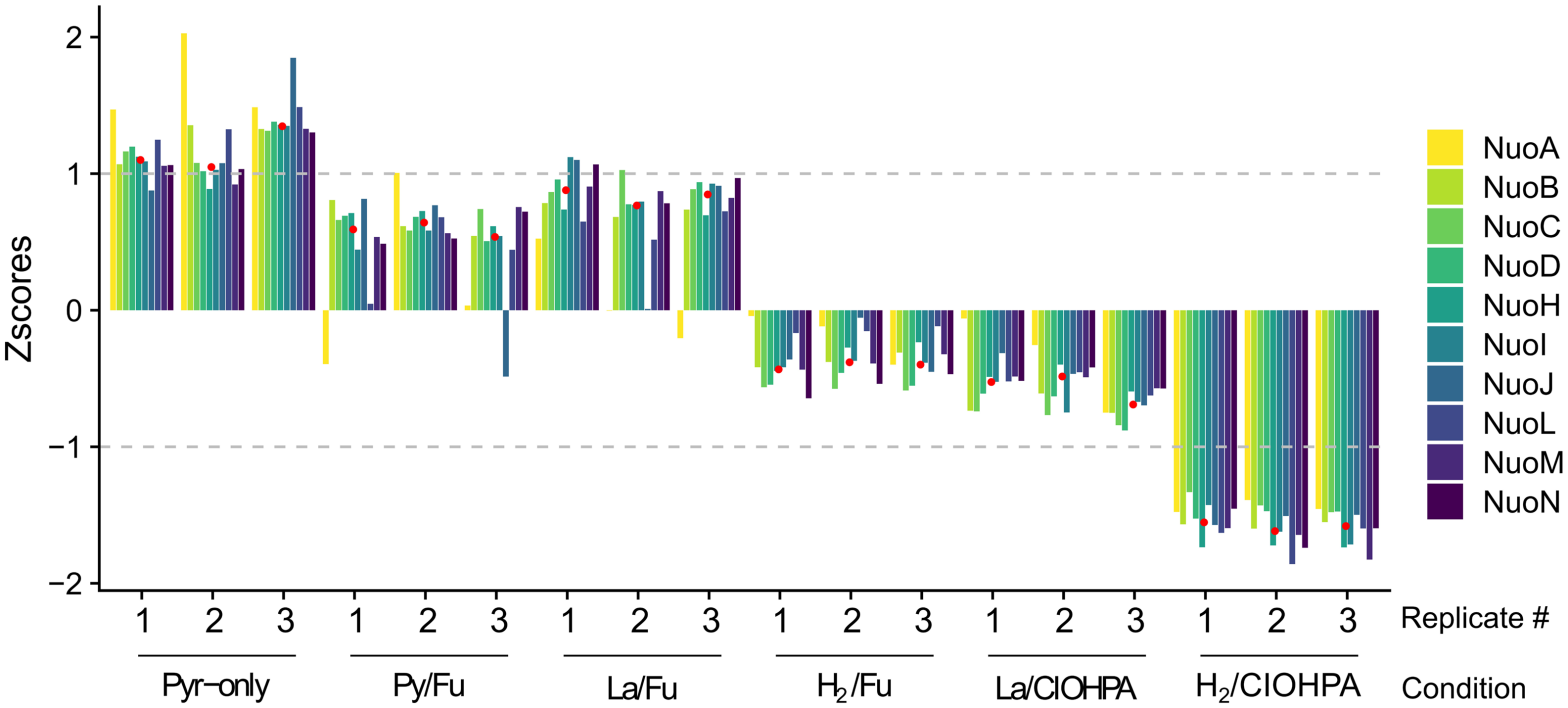
Relative abundance of 10 Nuo homologous subunits in *D. hafniense* strain DCB-2 across 6 different growth conditions run in triplicates. The Z-scores for each of the Nuo homologous proteins were calculated and displayed across the different conditions. The median of all Nuo homologues was calculated for each growth condition (indicated as red dots) and used to screen the proteomic dataset for possible partners of the complex I-like enzyme in strain DCB-2.

As the predominance of the complex I-like enzyme in one or the other growth conditions may reflect its metabolic relevance, the dataset obtained from the comparative proteomic analysis of strain DCB-2 in our previous study (Willemin et al., 2023) was used to calculate the relative abundance (expressed as Z-scores) of the Nuo homologous protein subunits across the six growth conditions (Figure 4). This result indicated that the abundance of the complex I-like enzyme did not vary drastically amongst all the samples and that all the scores were comprised between −1.5 and + 1. Nevertheless, as shown in Figure 4, a clear trend can be seen in the common behaviour of all the Nuo subunits. Indeed, except for the two subunits NuoA and NuoJ, which showed a noisier pattern in Z-scores, all other components of the complex were relatively more expressed in the growth conditions Py-only, Py/Fu, and La/Fu and less expressed in the condition H_2_/ClOHPA. In the conditions La/ClOHPA and H_2_/Fu, the Z-scores were slightly below 0, indicating that in these conditions, the level of expression of the complex was close to the average Z-score.

The median representing the typical behaviour of a Nuo protein was calculated (displayed as red dots in Figure 4) and was used to screen the ~3,000 proteins quantified in the proteomic analysis for those showing a similar profile of relative abundances and search for possible physiological protein partners of the complex I-like enzyme. All the proteins were ranked according to the Euclidean distance to the median and proteins with the smallest distance were considered as the most promising partners. A total of 47 proteins with an Euclidean distance smaller than 2.22 were identified (Supplementary Table S1). This threshold was selected based on the Euclidean distance of the most divergent Nuo subunit (NuoA with 2.21), whilst the distance of most of the other Nuo subunits ranged from 0.41 to 0.91 (Supplementary Table S1, indicated in bold). Within this selection, seven proteins harbouring redox cofactors could possibly be involved in electron transfer to the complex I-like enzyme (Table 1). Amongst them, the pyruvate ferredoxin oxidoreductase (PFOR, ACL18023) is the major candidate as an electron-donating enzyme since pyruvate oxidation to acetyl-CoA is very likely occurring in cells cultivated with pyruvate or lactate as the electron donor. A ferredoxin, such as ACL21378, which is also present in Table 1, is possibly shuttling electrons from PFOR to the complex I-like enzyme.

**Table 1:** FeS-containing proteins possibly involved in electron transfer to the complex I-like enzyme.

Accession	Description	Euclidean distance to Nuo subunits
ACL18123	Pyruvate flavodoxin/ferredoxin oxidoreductase	1,20
ACL19056	FeFe-hydrogenase	2,10
ACL20267	Rubredoxin-type protein	2,07
ACL20268^a^	Rubrerythrin	2,45
ACL20933^b^	Protein of unknown function DUF162	2,35
ACL20934	Protein of unknown function DUF224	1,18
ACL21378	[4Fe/4S] ferredoxin iron–sulfur binding domain protein	2,21
ACL21807	[4Fe/4S] ferredoxin iron–sulfur binding domain protein	1,96
ACL21808	Carbon monoxide dehydrogenase, catalytic subunit	2,10

a Despite its Euclidian distance beyond 2,22, ALC20268 was selected in the table as it is the probable functional partner of ACL20267.

b Despite its Euclidian distance beyond 2,22, ALC20933 was selected in the table as it is the probable functional partner of ACL20934.

Two additional redox enzymes, which are usually involved in energy metabolism, could also transfer electrons to the complex I-like enzyme. These are the cytoplasmic monomeric FeFe-hydrogenase (ACL19056) and the carbon monoxide dehydrogenase (CODH, ACL21807-8). Although CODH has been detected as upregulated during pyruvate fermentation previously (Willemin et al., 2023), its physiological role in the reduction of the quinone pools via the complex I-like enzyme is not clear. Finally, two proteins harbouring FeS clusters were also present in this selection (ACL20267 and ACL20934). The former, annotated as rubredoxin, could pair with its rubrerythrin partner (ACL20268, with a Euclidian distance of 2.45) and be involved in electron transfer in the context of oxidative stress, as reported earlier for *Desulfovibrio vulgaris* (Lumppio et al., 2001). ACL20934, with its partner protein ACL20933, which had a Euclidian distance of 2.35, is not functionally annotated in the genome but ACL20933 shows some similarity to the LutB subunit of lactate utilising enzyme, thus possibly involved in electron transfer from lactate to the complex I-like enzyme. Interestingly, the proteins initially annotated as NuoE (ACL19452) or NuoF (ACL19453) were found within the proteins that were the most distant from the median, further supporting the fact that they are not likely partners of the complex.

## Discussion

In this study, rotenone and, to a lesser extent, piericidin A were used as complex I inhibitors. With lactate and pyruvate as carbon and energy sources, *D. hafniense* strain DCB-2 did not grow in the presence of rotenone, whereas growth with H_2_ as an electron donor was not affected. These surprising results indicated that the complex I-like enzyme was mainly producing menaquinol, the direct electron donor to the terminal reductases of the anaerobic respiration chains tested here, either the fumarate reductase (Lancaster et al., 1999) or the reductive dehalogenase (Cimmino et al., 2023). The missing NADH-oxidising module suggests that there must be an alternative electron-donating protein to the complex I-like enzyme. In the following, we discuss first the potential redox partners of the complex I-like enzyme and second the physiological role of this complex in the energy metabolism of *D. hafniense* strain DCB-2.

### Potential redox partners of the complex I-like enzyme in *Desulfitobacterium hafniense* strain DCB-2

Respiratory complex I is well known for its function in the respiration of *E. coli*, although it is also predicted to be involved in various other bacterial physiological pathways (Spero et al., 2015). Based on the similar genetic organisation of the region encoding the *nuo* homologous genes, it is likely that complex I-like enzymes adopt a similar conformation in OHRB and other bacteria and thus might play a similar role. Indeed, except for the NADH-oxidising module, which may be replaced by an alternative electron donor, the important features for electrons channelling to the quinone reduction site and the formation of the proton pumping module are conserved.

In this study, the proteomic data published recently (Willemin et al., 2023) were used to identify potential electron donors of the complex I-like enzyme in *D. hafniense* strain DCB-2. Amongst the proteins which showed an expression pattern very close to the Nuo homologues across the 18 samples analysed by proteomics, 4 putative redox partners were identified. These proteins were either constituted by one single protein (ACL19056) or by a set of two proteins that are likely to interact with each other (ACL18123/ACL21378, ACL20267-8, and ACL21807-8). The fact that these proteins follow a similar trend as other Nuo homologues do not, by itself, prove that they directly interact with the complex I-like enzyme. Experimental data would be necessary to support one or the other scenario. Nevertheless, the proteins highlighted using the expression pattern approach represent interesting candidates and their hypothetical interaction with the complex I-like enzyme is further discussed below.

**Figure 5:**
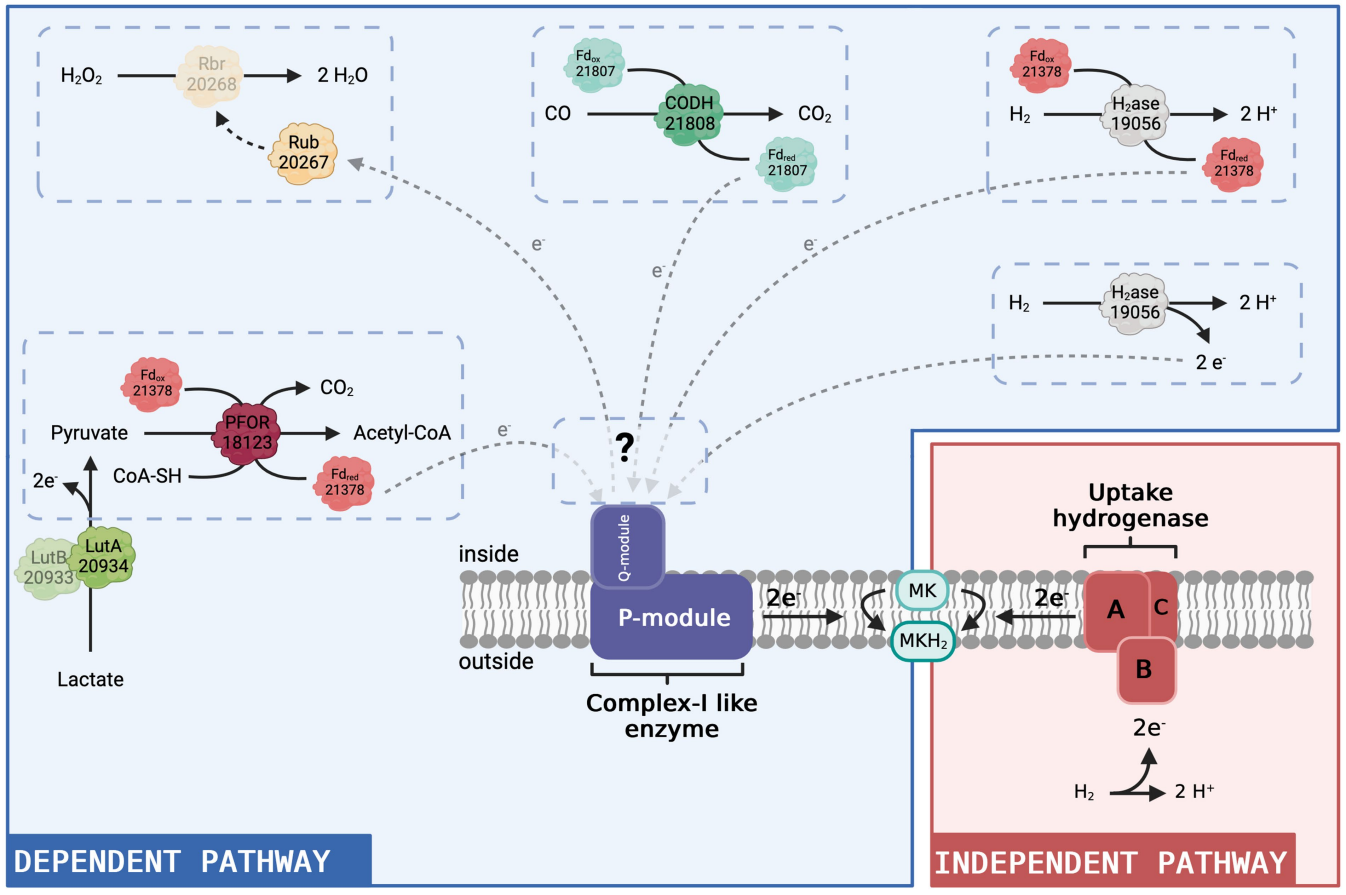
Hypothetical model of the complex I-like enzyme-dependent and -independent pathways to produce menaquinol in *D. hafniense* strain DCB-2. Given the results presented in this present study, we propose that the complex I-like enzyme serves as an entry point into the respiratory chain for electrons from the cytoplasm (blue panel). All four putative redox partners (constituted by a total of seven proteins) identified in the proteomic dataset are represented as direct or indirect donors to the complex I-like enzyme with their respective locus number (ACL#). In contrast, in respiration processes where the uptake hydrogenase is the electron-donating moiety (red panel), the electron donor (H_2_) delivers electrons directly to the menaquinone pool in the membrane from outside and there is no need for a cytoplasmic entry point. MK: menaquinone, MKH_2_: menaquinol. Figure created with BioRender.com.

ACL19056 has previously been identified as a cytoplasmic FeFe-hydrogenase (Willemin et al., 2023). It could either directly interact with the complex I-like enzyme or transfer electrons upon hydrogen oxidation via a ferredoxin (Figure 5). Interestingly, the NuoG subunit of the N-module in *E. coli* has been shown to display similarities with molybdopterin-containing proteins, such as FeFe hydrogenases (Moparthi and Hägerhäll, 2011), indicating that this FeFe hydrogenase could interact directly with the complex I-like enzyme. One could also imagine the involvement of other proteins to stabilise the interaction between the hydrogenase and the NuoI subunit, which contains the first redox cofactor of the Q-module and accepts electrons from the N-module. The possibility that the complex I-like enzyme receives electrons from a ferredoxin has already been described in cyanobacteria (Battchikova et al., 2011; Nowaczyk et al., 2012). However, both scenarios require the presence of intracellular H_2_. The latter could be produced by the Ech-type hydrogenase that is more abundant with lactate as an electron donor compared to H_2_ (Willemin et al., 2023).

Amongst the proteins with a similar expression pattern as the Nuo subunits, there are two ferredoxins, ACL21378 and ACL21807. ACL21807 is encoded directly upstream of the carbon monoxide dehydrogenase (CODH, ACL21808) and is therefore likely the electron-accepting protein of CODH oxidising CO to CO_2_. Neither ferredoxin-encoding gene is found in the direct vicinity of the FeFe-hydrogenase nor the pyruvate:ferredoxin oxidoreductase (PFOR, ACL18173) that appears as one of the potential redox partners of the complex I-like enzyme. In the model presented in Figure 5, ACL21378 was assigned as a possible redox partner of PFOR, transferring electrons from pyruvate oxidation to the complex I-like enzyme and/or the FeFe-hydrogenase discussed above. The cyanobacterial complex I-like enzyme using a ferredoxin displays additional subunits for efficient delivery of the electrons from the ferredoxin (Nowaczyk et al., 2012). The presence of additional subunits playing a similar role in the complex I-like enzymes from OHRB is not excluded but can only be verified by a thorough biochemical characterisation of the enzyme.

Pyruvate was present in many of the tested growth conditions and thus the activity of PFOR is largely supported. In contrast, carbon monoxide was not directly added to the culture medium. However, the oxidation of CO to CO_2_ coupled with the reduction of a ferredoxin could be due to CO formation from pyruvate by a yet unknown enzyme, as proposed for *Desulfovibrio vulgaris* Hildenborough (Voordouw, 2002). ACL21808 and its associated ferredoxin show a high sequence identity with CODH I (CooSI, 70%) and CooF3 (50%) of *Carboxydothermus hydrogenoformans*. In the latter bacterium, the electrons supplied by CODH I are used for the translocation of protons through the cytoplasmic membrane by a complex I-related hydrogenase (Svetlitchnyi et al., 2001). CODH activity thus remains an interesting candidate for feeding electrons to the complex I-like enzyme in strain DCB-2 (Figure 5).

The last potential redox partner for the complex I-like enzyme highlighted in the proteomic data is a set of proteins composed of rubrerythrin and rubredoxin (ACL20267-8). In *Desulfovibrio vulgaris*, homologous proteins are found to be involved in a protective mechanism against oxidative stress (Lumppio et al., 2001). Rubredoxin receives electrons from an undefined source, which, in our case, could be the complex I-like enzyme, and transfers them to the rubrerythrin moiety, which performs the reduction of H_2_O_2_ to H_2_O (Lumppio et al., 2001). This implies that the complex I-like enzyme works in the reverse direction, using the proton gradient to transfer electrons from menaquinol to rubredoxin (Figure 5). Consuming energy for protection against oxidative stress might be a necessity in strict anaerobes such as OHRB. Furthermore, this proposition somehow recalls the mechanism of ROS generation by mitochondrial complex I, where, in contrast to our hypothesis, electrons delivered by complex I represent a source of oxidative stress.

### The physiological relevance of complex I-like enzymes in Firmicutes OHRB

Globally, the results obtained from the growth inhibition experiments with complex I-specific inhibitors such as rotenone and piericidin highlighted what seems like a fundamental difference between conditions involving H_2_ or lactate and pyruvate as electron donors. Indeed, bacterial growth was totally inhibited by the addition of rotenone when *D. hafniense* strains DCB-2 and TCE1 were cultivated in respiratory or fermentative conditions with lactate or pyruvate as the primary carbon and energy sources. The effect observed after treating *D. hafniense* strain DCB-2 cultures growing in fermentative condition and in fumarate respiration using lactate as electron donor were less drastic with piericidin A than with rotenone but still showed a significant reduction of growth in response to the presence of the inhibitor. This latter observation might be explained by a reduced affinity of the complex I-like enzyme for piericidin. A higher concentration of piericidin A could be tested to evaluate whether it would result in a more drastic growth reduction. Nevertheless, in contrast to what is described above for growth conditions involving lactate or pyruvate as an electron donor, the inhibition of the complex I-like enzyme with both inhibitors had no effect on the growth of *D. hafniense* strain DCB-2 in H_2_/ClOHPA or H_2_/Fu, nor did rotenone affect the growth of *D. restrictus* in H_2_/PCE. Although *D. hafniense* strain TCE1 in H_2_/PCE showed a slight growth defect when treated with rotenone, those results suggest that OHRB from the genus *Desulfitobacterium* mainly rely on complex I-like enzymes for growth except when using H_2_ as an electron donor.

From that observation and as displayed in Figure 5, we propose that the complex I-like enzyme is necessary for the electrons to enter the respiratory chain only when the source of electrons is located in the cytoplasm. In contrast, in respiration processes where the uptake hydrogenase is the electron-donating moiety, the electron donor (H_2_) delivers them directly to the menaquinone pool in the cytoplasmic membrane and there is no need for a cytoplasmic entry point. Although none of the six expressed hydrogenases was systematically detected as upregulated with H_2_ as an electron donor, an H_2_-uptake NiFe-hydrogenase was more expressed when H_2_ was present (Willemin et al., 2023). In the case where the electrons originate from electron donor oxidation in the cytoplasm, we propose that the electrons are delivered to the complex I-like enzyme, either directly by the cytoplasmic FeFe hydrogenase or by reduced ferredoxins produced by PFOR or FeFe-hydrogenase, as described above. The interactions of the complex I-like enzyme with several electron donors, however, are not excluded. Considering that, one possible explanation for the slight reduction of growth induced by rotenone observed for strain TCE1 in H_2_/PCE conditions could be that this strain uses both a membrane-bound uptake hydrogenase and a cytoplasmic hydrogenase for H_2_ oxidation. The partial growth defect would then correspond to the inhibition of the cytoplasmic pathway, whilst the one attached to the membrane still provides sufficient energy for growth, but at a lower rate.

One aspect which remains to be discussed is the special case of fermentation. Indeed, when *D. hafniense* strain DCB-2 was cultivated in pyruvate-only conditions, a clear growth defect was observed in response to the rotenone treatment. This came as a surprise, as the involvement of complex I-like for the reduction of menaquinones was not expected in the absence of any terminal acceptor. However, a resurgence of growth was observed after 30 h of incubation. This observation remains difficult to explain but could be the result of two possible metabolic pathways. First, a progressive accumulation of H_2_ could emerge from fermentative activity, leading to its use as an electron donor as presented above, thus bypassing the complex I-like enzyme. A second plausible explanation could be the use of sulfite as an electron acceptor when strain DCB-2 grows on pyruvate-only, as discussed earlier (Willemin et al., 2023). Indeed, in our previous studies, we showed that the dissimilatory sulfite reduction (DSR) pathway was upregulated in pyruvate-only conditions, suggesting that small amounts of sulfite could emerge in the culture medium by the chemical oxidation of sulfide, which is supplemented as a reducing agent in the preparation of the anaerobic medium. Therefore, the pyruvate-only conditions are considered rather as “pseudo”-fermentative, facilitated sulfite reduction. Here, the delay of growth observed for cells in pyruvate-only conditions treated with rotenone could reflect a switch in metabolism. In the initial phase, the rotenone inhibits the complex I-like enzyme involved in recovering electrons from sulfite reduction, and then later true fermentation is activated, which does not need the quinone pool or the complex I-like enzyme.

## Data availability statement

Publicly available datasets were analyzed in this study. The data is available in the ProteomeXchange Consortium (www.ebi.ac.uk/pride; with accession number: PXD030393).

## Author contributions

MSW: Conceptualization, Data curation, Formal analysis, Investigation, Methodology, Project administration, Validation, Visualization, Writing – original draft, Writing – review & editing. FA: Data curation, Formal analysis, Methodology, Validation, Visualization, Writing – review & editing. RH: Methodology, Validation, Writing – review & editing. JM: Conceptualization, Formal analysis, Funding acquisition, Methodology, Project administration, Supervision, Validation, Visualization, Writing – original draft, Writing – review & editing. CH: Conceptualization, Funding acquisition, Supervision, Writing – review & editing.
