# Supplementary material for: Conditional essentiality of the 11-subunit complex I-like enzyme in strict anaerobes: the case of Desulfitobacterium hafniense strain DCB-2
Source: Front Microbiol. 2024 Jun 26;15:1388961. doi: 10.3389/fmicb.2024.1388961 (PMC11238625; doi:10.3389/fmicb.2024.1388961)
Supplement: Supplementary file 1 [file Data_Sheet_1.pdf]

Supplementary material to :

Conditional essentiality of the 11-subunit complex I-like enzyme in strict anaerobes: the case of *Desulfitobacterium hafniense* strain DCB-2

Mathilde Stéphanie Willemin[1#], Florence Armand[2], Romain Hamelin[2], Julien Maillard[1] and Christof Holliger[1]

[1] Laboratory for Environmental Biotechnology (LBE), Ecole Polytechnique Fédérale de Lausanne (EPFL), Lausanne, Switzerland

[2] Proteomic Core Facility (PCF), Ecole Polytechnique Fédérale de Lausanne (EPFL), Lausanne, Switzerland

# corresponding author: mathilde.willemin@epfl.ch, present address[2]

Table S1. Proteins displaying a Euclidian distance < 2.22 to relative abundance profile of the complex I-like subunits.

Accession	Euclidean Distance	Protein annotation
ACL21016	0,40	Hypothetical protein
**ACL21758**	**0,42**	**NuoM**, proton-translocating NADH-quinone oxidoreductase, chain M
**ACL21764**	**0,43**	**NuoD**, NADH dehydrogenase (quinone)
**ACL21762**	**0,45**	**NuoI**, 4Fe/4S ferredoxin iron-sulfur binding domain protein
**ACL21763**	**0,47**	**NuoH**, NADH dehydrogenase (quinone)
**ACL21757**	**0,48**	**NuoN**, proton-translocating NADH-quinone oxidoreductase, chain N
**ACL21766**	**0,50**	**NuoB**, NADH-quinone oxidoreductase, B subunit
**ACL21765**	**0,68**	**NuoC**, NADH dehydrogenase (ubiquinone) 30 kDa subunit
**ACL21759**	**0,91**	**NuoL**, proton-translocating NADH-quinone oxidoreductase, chain L
ACL20934*	1,18	Protein of unknown function, DUF224
ACL18123*	1,20	Pyruvate flavodoxin/ferredoxin oxidoreductase
ACL18537	1,22	2-succinyl-6-hydroxy-2,4-cyclohexadiene-1-carboxylic acid synthase/2-oxoglutarate decarboxylase
ACL22171	1,43	HEPN domain protein
**ACL21761**	**1,49**	**NuoJ**, NADH-ubiquinone/plastoquinone oxidoreductase chain 6
ACL18536	1,55	Isochorismate synthase
ACL22578	1,58	Conserved hypothetical protein
ACL18461	1,62	2-C-methyl-D-erythritol 4-phosphate cytidylyltransferase
ACL21302	1,64	Protein of unknown function, DUF815
ACL18389	1,65	Transcriptional regulator, AbrB family
ACL20854	1,67	ABC transporter related
ACL18419	1,69	Carbamoyl-phosphate synthase, large subunit
ACL19215	1,74	Sodium/proline symporter
ACL21908	1,75	Phosphopantothenoylcysteine decarboxylase/phosphopantothenate/cysteine ligase
ACL22086	1,80	Beta-galactosidase/beta-glucuronidase-like protein
ACL22030	1,82	Uracil phosphoribosyltransferase
ACL20852	1,86	Protein of unknown function, DUF534
ACL22847	1,90	Glutamate dehydrogenase
ACL20476	1,91	GCN5-related N-acetyltransferase
ACL18335	1,95	Ribonucleoside-diphosphate reductase, adenosylcobalamin-dependent
ACL20442	1,96	Acetyl-CoA carboxylase, carboxyl transferase, alpha subunit
ACL21807*	1,96	4Fe/4S ferredoxin iron-sulfur binding domain protein
ACL19598	1,99	Conserved hypothetical protein
ACL19658	1,99	Methyl-accepting chemotaxis sensory transducer
ACL22058	2,01	Hypothetical protein
ACL22579	2,03	D-isomer specific 2-hydroxyacid dehydrogenase
ACL18334	2,03	ATP-cone domain protein
ACL22184	2,04	Glycosyl transferase group 1
ACL19545	2,05	Histidinol-phosphate aminotransferase
ACL20267*	2,07	Rubredoxin-type protein
ACL20958	2,08	Hypothetical protein
ACL21570	2,09	Hypothetical protein
ACL19577	2,09	Sodium/solute symporter
ACL19056*	2,10	Hydrogenase large subunit domain protein
ACL21808*	2,10	Carbon-monoxide dehydrogenase, catalytic subunit
ACL20971	2,11	Aminotransferase class I and II
ACL18386	2,12	Transcriptional regulator, CdaR
ACL21728	2,13	Heat shock protein, ATPase subunit
ACL22215	2,16	Citrate lyase, acyl carrier protein
ACL20803	2,16	Conserved hypothetical protein
ACL19141	2,19	Threonine aldolase
ACL21115	2,19	GCN5-related N-acetyltransferase
ACL22109	2,20	Cell envelope-related transcriptional attenuator
ACL18097	2,20	Conserved hypothetical protein
**ACL21767**	**2,21**	**NuoA**, NADH-ubiquinone/plastoquinone oxidoreductase chain 3
ACL20965	2,21	Thioesterase superfamily protein
ACL19574	2,21	Homocitrate synthase
ACL21378*	2,21	4Fe/4S ferredoxin iron-sulfur binding domain protein

In bold are indicated the homologous Nuo subunits
* Candidate protein partners of the complex-I like enzyme (see Table 1)

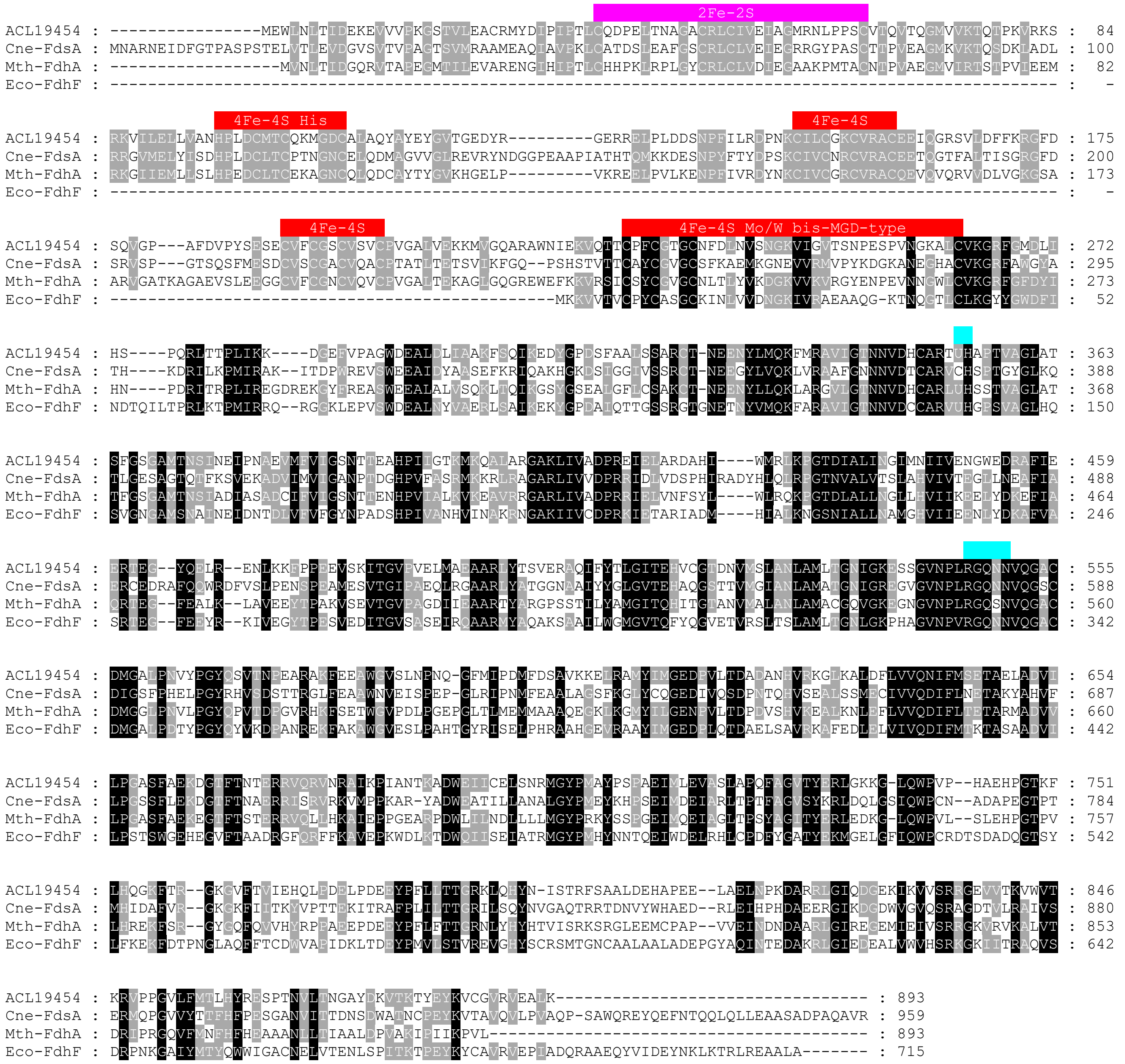

Identity scores

	ACL19454	Cne-FdsA	Mth-FdhA	Eco-FdhF
ACL19454	100%	39%	52%	32%
Cne-FdsA	-	100%	38%	27%
Mth-FdhA	-	-	100%	30%
Eco-FdhF	-	-	-	100%

Figure S1. (A) Sequence likelihood analysis of *D. hafniense* ACL19454 with selected members of the catalytic unit of class I formate dehydrogenases (Arias-Cartin et al, 2022). In contrast to FdhF from *E. coli* (Eco), but similarly to FdsA from *Cupriavidus necator* (Cne) and FdhA from *Moorella thermoacetica* (Mth), the homologue in *D. hafniense* strain DCB-2 shows an extended N-terminal region. Conserved cysteine motifs binding 2Fe-2S and 4Fe-4S clusters are shown in pink and red, respectively, while blue boxes indicate the conserved amino acid of the FDH active site. The sequence alignment was done with ClustalX2 (Larkin et al., 2007). Accession numbers: Cne-FdsA, SCU75130; Mth-FdhA, P77908, Eco-FdhF, P07658.

```
                                                                2Fe-2S
ACL19454 : -------------MEWLNLTIDEKEVVVPKGSTVLEACRMYDIPIPTLCQDPELTNAGACRLCIVEIAG-----MRNLPPSCVTQVTQGMVVKTQTP--K :  80
Eco-NuoG : ---------------MATIHVDGKEYEVNGADNLLEACLSLGLDIPYFCWHPALGSVGACRQCAVKQYQNAEDTRGRLVMSCMTPASDGTFISIDDE--E :  83
Smu-NuoG : -------------MSDVLITIDGKQCTATEGEYVLGVARRNDIFIPALCYVTNCSPTLACRLCLVDIDG-------KRAYSCNARAKEGMNVITKTE--E :  78
Mtu-NuoG : MTQAADTDIRVGQPEMVTLTIDGVEISVPKGTLVIRAAELMGIQIPRFCDHPLLEPVGACRQCLVEVEG-----QRKPLASCTTVATDDMVVRTQLTSEI :  95

                     4Fe-4S His                                          4Fe-4S
ACL19454 : VRKSRKVILELLVANHPLDCMTCQKMGDCALAQYAYEYGVTGEDYRGERRELPLDD-SNPFILRDPNKCILCGKCVRACEEIQGRSVLDFFKRGFDSQVG : 179
Eco-NuoG : AKQFRESVVEWLMTNHPHDCPVCEEGGNCHLQDMTVMTGHSFRRYRFTKRTHRNQD-LGPFISHEMNRCIACYRCVRYYKDYADGTDLGVYGAHDNVYFG : 182
Smu-NuoG : IEKERRAIMEIYDINHPLECGVCDQSGECELQNYTLHMAVDSQHHCIADTHRPTKQ--WGKIHYDASLCIVCERCVTVCKDMIGESALKTVPRGGAELDK : 176
Mtu-NuoG : ADKAQHGVMELLLINHPLDCPMCDKGGECPLQNQAMSNGRTDSRFTEAKRTFAKPINISAQVLLDRERCILCARCTRFSDQIAGDPFIDMQERGALQQVG : 195

                                                       4Fe-4S Mo/W bis-MGD-type
ACL19454 : PAFDVPYSESECVFCGSCVSVCPVGALVEKKMVGQ-ARAWNIEKVQTTCPFCGTGCNFDLNVSNGKVIGVTSNPESPVNGKALCVKGRFGMDLIHSPQRL : 278
Eco-NuoG : RPEDGTLES---EFSGNLVEICPTGVFTDKTHSERYNRKWDMQFAPSICQQCSIGCNISPGERYGELRRIENRYNGTVNHYFLCDRGRFGYGYVNLKDRP : 279
Smu-NuoG : AWKDKTEKD--AYAMWNKLQKSIIGIASG-------AETLDCTQCGECTAVCPVGALVSSDFQYTSNAWELQKIPASNPHSSDCSLLYYDVKHTSTSNPE : 267
Mtu-NuoG : IYADEPFES---YFSGNTVQICPVGALTGTAYRFR-ARPFDLVSSPSVCEHCASGCAQRTDHRRGKVLRRLAGDDPEVNEEWNCDKGRWAFTYATQPDVI : 291

ACL19454 : TTPLIKKDGE----FVPAG------WDEALDLIAAKFSQIKEDYGPDSFAALSSARCTNEENYLMQKFMRAVIGTNNVDHCARTUHAPTVAGLATSFGSG : 368
Eco-NuoG : RQPVQRRGDD----FITLN------AEQAMQGAADILRQSKKVIG------IGSPRASVESNFALRELVGEENFYTGIAHGEQERLQLALKVLRE---GG : 360
Smu-NuoG : PKIYRVNSDHNYAPLHAAARYGFDFQNEVTCKDESAFARVVELLQTKVDTIVFDSYITNEEALILQKLKEKFG--FKLVNEDAKAYQNFLKHFASTSGSN : 365
Mtu-NuoG : TTPLIRDGGDPKGALVPTS------WSHAMAVAAQGLAAARGRTG-----VLVGGRVTWEDAYAYAKFARITLGTNDIDFRARPHSAEEADFLAARIAGR : 380

ACL19454 : AMTNSINEIPNAEVMFVIGSNTTEAHPIIGTKMKQALARG--AKLIVADPREIELARDAHIWMRLKPGTDIALINGIMNIIVENGWEDRAFIEERTEGYQ : 466
Eco-NuoG : IYTPALREIESYDAVLVLGEDVTQTGARVALAVRQAVKGKAREMAAAQKVADWQIAAILNIGQRAKHPLFVTN--------------------------- : 433
Smu-NuoG : LYSGDLKFISESNFVVSFGSAIKTDSPNAGYAMNNAIGMNKGAGLYFHPVADPIVSAYSKNLLSITHKIG------------------------------ : 435
Mtu-NuoG : HMAVSYADLESAPVVLLVGFEPEDESPIVFLRLRKAARRHRVPVYTIAPFATGGLHKMSGRLIKTVPGGE------------------------------ : 450

ACL19454 : ELRENLKKFPPEEVSKITGVPVELMAEAARLYTSVERAQIFYTLGITEHVCGTDNVMSLANLAMLTGNIGKESSGVNPLRGQNNVQGACDMG-ALPNVYP : 565
Eco-NuoG : VDDTRLDDIAAWTYRAPVEDQARLGFAIAHALDNSAPAVDG---------------IEPELQSKIDVIVQALAGAKKPLIISGTNAGSLEVIQAAANVAK : 518
Smu-NuoG : -AEEAIAYLLLDLFGDKAAMPKGVVDYLASFHAVEKKIIQES-------VKEEIKEMVKDEESGEEKEVVKTITKMVETEIEVDTNALLEMIGEEVDLVE : 527
Mtu-NuoG : --PAALDDLATGAVGDLLATPGAVIIVGERLATVPG---------------------GLSAAARLADTTGARLAWVPRRAGE---RGALEAG-ALPTLLP : 523

ACL19454 : GYQSVTNPEARAKFEEAWGVSLNPNQGFMIPDMFDSAVKKELRAMYIMGEDPVLTDADANHVRKGLKALDFLVVQNIF-MSETAELADVILPGASFAEKD : 664
Eco-NuoG : ALKGRGADVGITMIARSVNSMGLGIMGGGSLEEALTELETGRADAVVVLENDLHRHASAIRVNAALAKAPLVMVVDHQ-RTAIMENAHLVLSAASFAESD : 617
Smu-NuoG : KITKLLDKKDSFSLIAGEDLYTHPRAEN--IAKLLGLIERFTAFKLVIIPSRTNTLGISLICDLDDVKGNFSLGYNVKGDFTLSSLGKGDLDMPALNQQE : 625
Mtu-NuoG : GGRPLADEVARAQVCAAWHIAELPAAAGRDADGILAAAADETLAALLVGGIEPADFADPDAVLAALDATGFVVSLELR-HSTVTERADVVFPVAPTTQKA : 622

ACL19454 : GTFTNTERRVQRVNRAIK--PIANT---KADWEIICELSNRMG--------YPMAYPSPAEIMLEVASLAPQFAGVTY----ERLGKKGLQWPVPHAEHP : 747
Eco-NuoG : GTVINNEGRAQRFFQVYDPAYYDSKTVMLESWRWLHSLHSTLLSREVDWTQLDHVIDAVVAKIPELAGIKDAAPDATFRIRGQKLAREPHRYSGRTAMRA : 717
Smu-NuoG : GTFTSMNKCVVPTNAALAFKGYCLN-----DIANAVGLEAEWT------IDYTPYLPSEKGFKKEKFDTLPN----------------RFENDGTENRGY : 698
Mtu-NuoG : GAFVNWEGRYRTFEPALRGSTLQAG---QSDHRVLDALADDMG--------VHLGVPTVEAAREELAALG--------------------IWDGKHAAGP : 691

ACL19454 : GTKFLHQGKFTRGKGVFTVIEHQLPD-------------------------------------------ELPDEEYPFLLTTGRKLQHYNISTRFS---- : 800
Eco-NuoG : NISVHEPRQPQDIDTMFTFSMEGNNQPTAHRSQVPFAWAPGWNSPQAWNKFQDEVGGKLRFGDPGVRLFETSENGLDYFTSVPARFQPQDGKWRIAPYYH : 817
Smu-NuoG : ALEPQTFTCKDNVEPIASFTCKEGDVVYR----------------------------------------ANPVYQFSAFTNKAHQLS------------- : 745
Mtu-NuoG : HIAATGPTQPEAGEAILTGWRMLLDE-------------------------------------------GRLQDGEPYLAGTAR---------------- : 732

ACL19454 : -----------AALDEHAPEELAEINPKDARRLGIQDGEKIKVVSRRGEVVTKVWVTKRVPPGVLFMTLHYRESPTNVLTNGAYDKVTKTYEYKVCGVRV : 889
Eco-NuoG : LFGSDELSQRAPVFQSRMPQPYIKLNPADAAKLGVNAGTRVSFSYDGNTVTLPVEIAEGLTAGQVGLPMGMSG-IAPVLAGAHLEDLKEAQQ-------- : 908
Smu-NuoG : ------------------EAGALYASTAFLEAKNLHDGAVVTVENEAGAKLVIAVKEEKMIDGLIAYLPTFDTKIDTAPFFKEGYRFAHVVIKGVEHV-- : 825
Mtu-NuoG : -------------------TPVVRLSPDTAAEIGAADGEAVTVSTSRGSITLPCSVTD-MPDRVVWLPLNSAGSTVHRQLRVTIGSIVKIGAGS------ : 806

ACL19454 : EALK : 893
Eco-NuoG : ---- :   -
Smu-NuoG : ---- :   -
Mtu-NuoG : ---- :   -
```

Identity scores

	ACL19454	Eco-NuoG	Smu-NuoG	Mtu-NuoG
ACL19454	100%	16%	13%	24%
Eco-NuoG	-	100%	10%	19%
Smu-NuoG	-	-	100%	15%
Mtu-NuoG	-	-	-	100%

Figure S1. (B) Sequence likelihood analysis of ACL19454 with selected members of the NuoG family. ACL19454 from *D. hafniense* strain DCB-2 was compared to NuoG homologues from *E. coli* (Eco), *Sulfurospirillum multivorans* (Smu) and *Mycobacterium tuberculosis* (Mtu). Here, only the cysteine motifs binding FeS clusters are conserved across the sequence selection (as shown in pink and red). The last 4Fe-4S cluster (annotated N7 in respiratory complex I) is in direct contact to a purine nucleoside triphosphate that resembles the GTP-derived molybdenum cofactor in formate dehydronases as shown in mycobacterial NuoG enzymes (Liang et al., 2023). The sequence alignment was done with ClustalX2 (Larkin et al., 2007). Accession numbers: Eco-NuoG, P33602; Smu-NuoG, AHJ11483; Mtu-NuoG, P9WIV9.

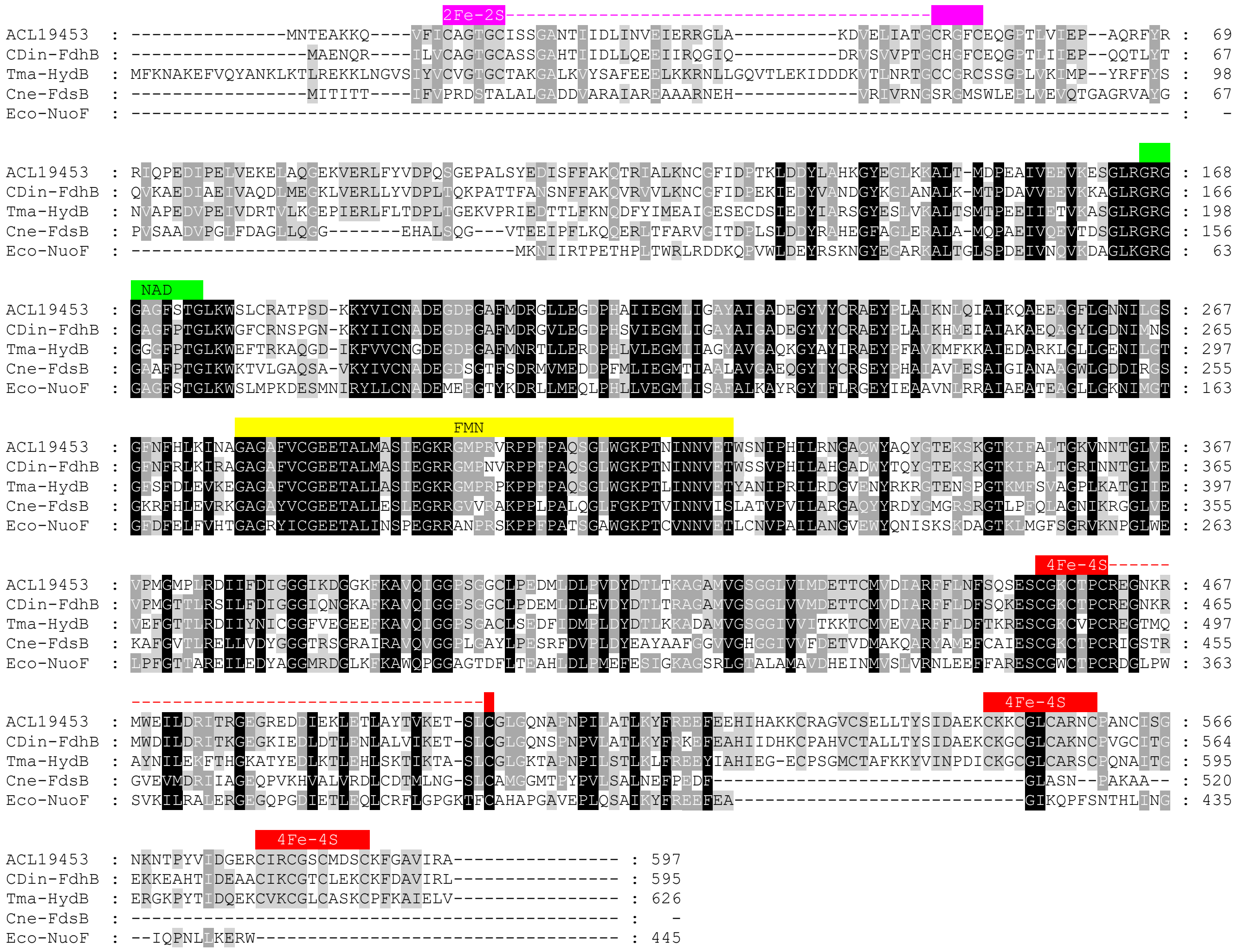

Identity scores

	ACL19453	CDin-FdhB	Tma-HydB	Cne-FdsB	Eco-NuoF
ACL19453	100%	72%	51%	38%	33%
CDin-FdhB	-	100%	49%	37%	30%
Tma-HydB	-	-	100%	34%	27%
Cne-FdsB	-	-	-	100%	31%
Eco-NuoF	-	-	-	-	100%

Figure S1. (C) Sequence likelihood analysis of ACL19453 with selected proteins. ACL19453 from *D. hafniense* strain DCB-2 was compared to FdhB from *Candidatus* Desulfosporosinus infrequens (CDin), HydB from *Thermotoga maritima* (Tma), FdsB from *Cupriavidus necator* (Cne) and NuoF from *E. coli* (Eco). In contrast to *E. coli* NuoF, ACL19453 and the other proteins display an extended N-terminus harboring a 2Fe-2S cluster binding motif (indicated in pink). A C-terminal motif for binding a 4Fe-4S cluster is shared across some members but absent in *E. coli* NuoF. The other features are conserved throughout the sequence selection (NAD-binding region in green, FMN-binding region in yellow and the motifs for two additional 4Fe-4S clusters). The sequence alignment was done with ClustalX2 (Larkin et al., 2007). Accession numbers: CDin-FdhB, SPF47987; Tma-HydB, O52682; Cne-FdsB, SCU75129; Eco-NuoF, A0A5B9A8I1.

```
                                                                                                              2Fe-2S---
ACL19452 : ------MCEECKELVDPKEEQLEQILAHHKREKGALIPVLQEAQGLYGYLPEHVIKHISRGLGIPSAKVYGVVTFYAQFRLTPMGRNVISVCLGTACHVR :  94
Tma-HydC : --------------MERHFEKVEEILKKYGYKRENLIKILLEIQEIYRYLPEDVINYVSTAMGIPPAKIYGVATFYAQFSLKPKGKYTIMVCDGTACHMA :  86
Cne-FdsG : -----MPEISPHAPASADATRIAAIVAARQDMPGALLPILHEIQDTQGYIPDAAVPVIARALNLSRAEVHGVITFYHHFRQQPAGRHVVQVCRAEACQSV :  95
Eco-NuoE : MHENQQPQTEAFELSAAEREAIEHEMHHYEDPRAASIEALKIVQKQRGWVPDGAIHAIADVLGIPASDVEGVATFYSQIFRQPVGRHVIRYCDSVVCHIN : 100

           ---------------------------------
ACL19452 : GGAKVLEAIEKDTKIKDGQTTEDGRFTLEIVNCIGACGLAPVMSINGNVHGRLNADQIPGILAEYK--------------- : 160
Tma-HydC : GSPEVLKAIEEETGLTPGNVTEDLMFSLDQVGCLGACALAPVMVINGEVYGNLTADKVKEILRKIKEKERESANV------ : 161
Cne-FdsG : GAEALAEHAQRALGCGFHETTADGQVTLEPVYCLGQCACGPAVMVGEQLHGYVDARRFDALVRSLRESSAEKTTEAVEAQA : 176
Eco-NuoE : GYQGIQAALEKKLNIKPGQTTFDGRFTLLPTCCLGNCDKGPNMMIDEDTHAHLTPEAIPELLERYK--------------- : 166
```

Identity scores

	ACL19452	Tma-HydC	Cne-FdsG	Eco-NuoE
ACL19452	100%	44%	27%	37%
Tma-HydC	-	100%	22%	26%
Cne-FdsG	-	-	100%	25%
Eco-NuoE	-	-	-	100%

Figure S1. (D) Sequence likelihood analysis of ACL19452 with selected proteins. ACL19452 from *D. hafniense* strain DCB-2 was compared to HydC from *Thermotoga maritima* (Tma), FdsG from *Cupriavidus necator* (Cne) and NuoE from *E. coli* (Eco). The conserved 2Fe-2S motif is indicated in pink. The sequence alignment was done with ClustalX2 (Larkin et al., 2007). Accession numbers: Tma-HydC, O52681; Cne-FdsG, SCU75128; Eco-NuoE, AAC75345.

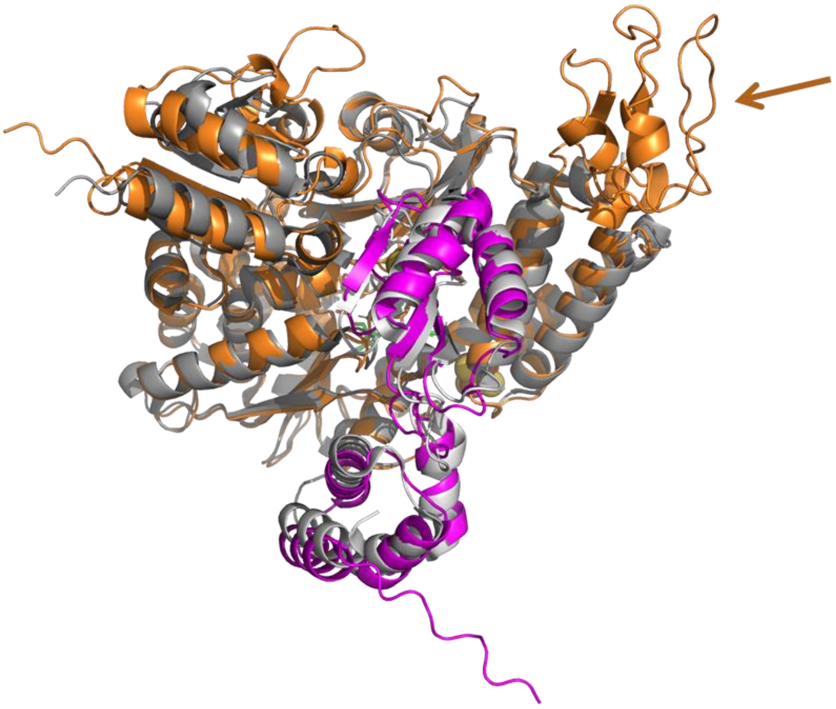

Figure S1. (E) Structure alignment of *D. hafniense* ACL19542-3 with *C. necator* FdsBG (PDB 6VW7). FdsB and FdsG are depicted in light and dark gray, respectively. ACL19452 is shown in magenta, while ACL19453 is in orange. Please note a C-terminal extension in ACL19453 (indicated by the arrow) that is likely to harbour FeS clusters. The structures of *D. hafniense* ACL19452-3 were obtained from AlphaFold DB (Jumper et al., 2021).

Figure S2

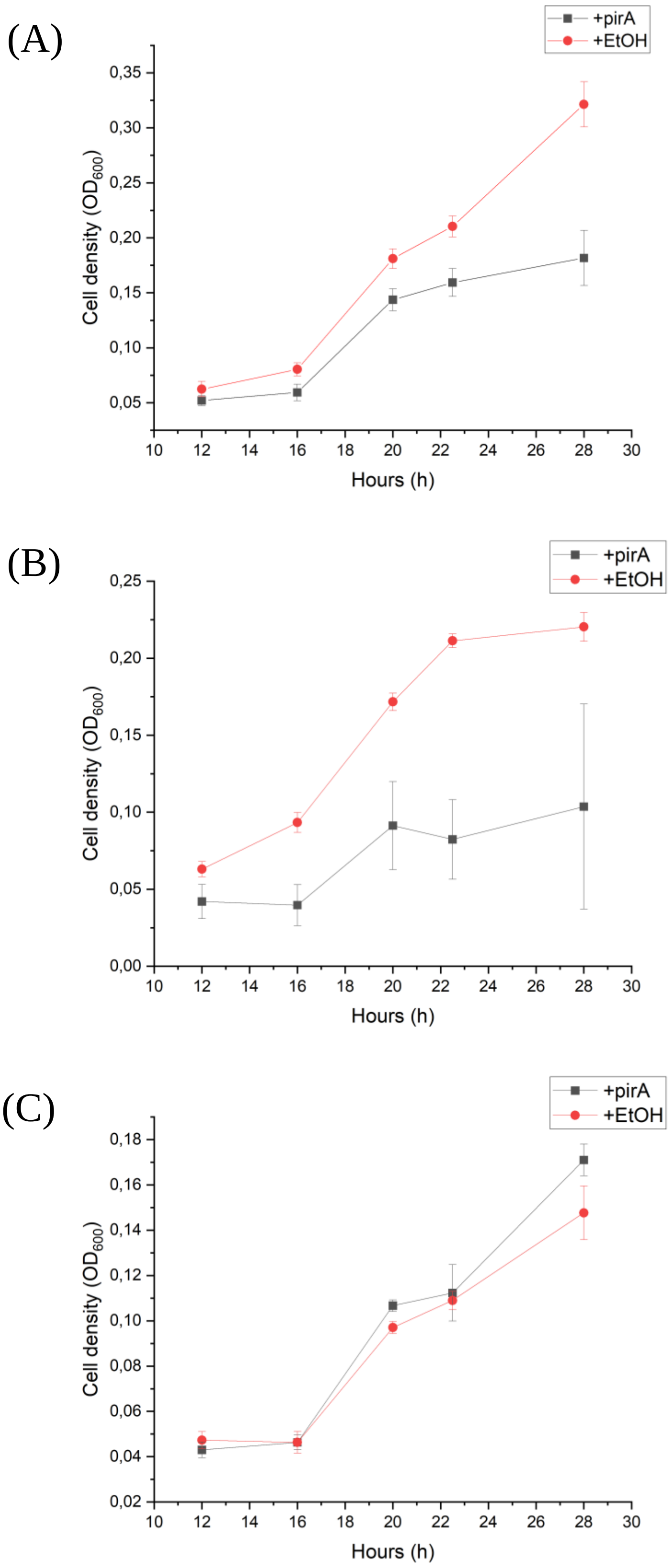

Figure S2. Growth curve of *D. hafniense* strain DCB-2 cultivated in the following conditions: (A) Pyruvate-only; (B) Lactate/Fumarate; (C) Hydrogen/Fumarate. For each condition, triplicates of two different cultures were performed with supplementation of piericidin A (+pirA) or ethanol (+EtOH).

Figure S3

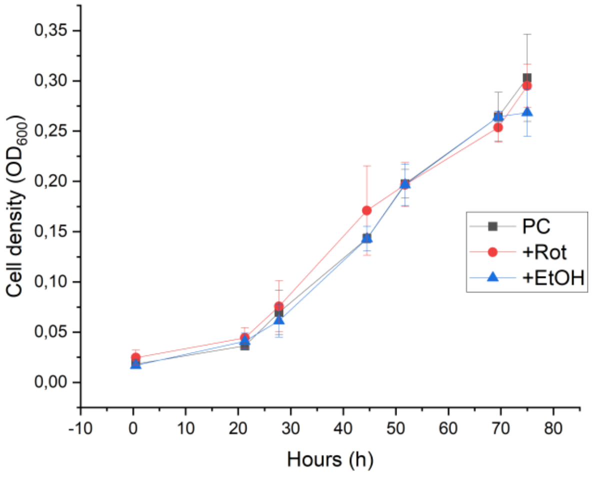

Figure S3. Growth curve of *Dehalobacter restrictus* strain PER-K23 cultivated with hydrogen/PCE. Triplicates of three different cultures were performed: positive control (PC), with supplementation of rotenone (+Rot), and of ethanol (+EtOH).

Figure S4

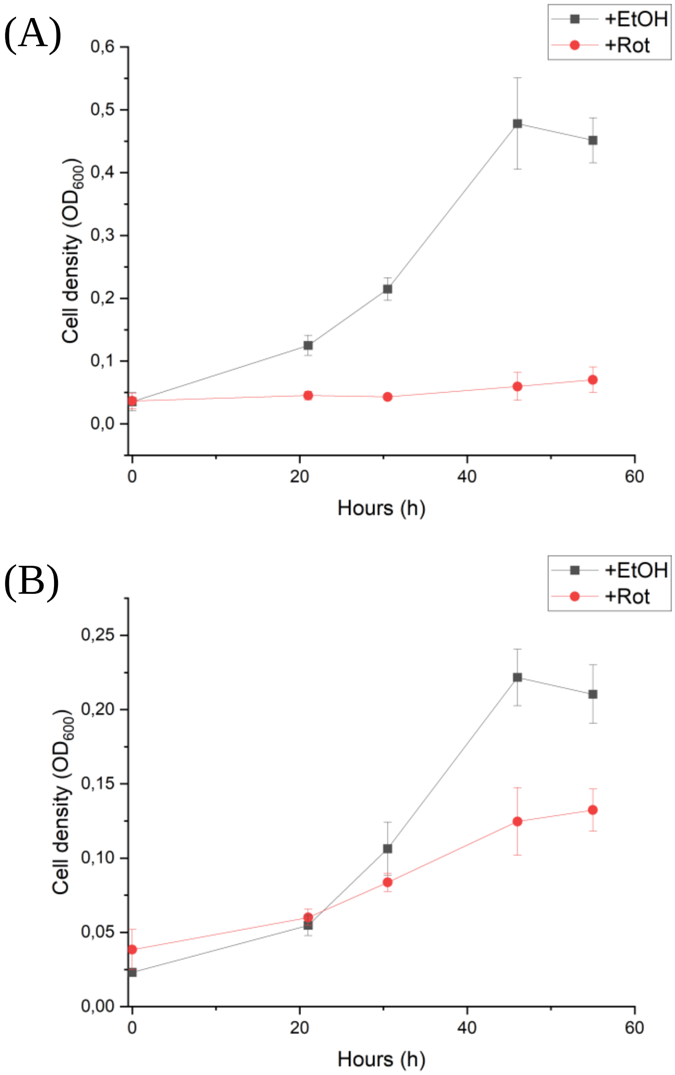

Figure S4. Growth curve of *D. hafniense* strain TCE1 cultivated in the following conditions: (A) Lactate/PCE; (B) Hydrogen/PCE. For each condition, triplicates of two different cultures were performed with supplementation of rotenone (+Rot) or ethanol (+EtOH).

Figure S5

(A)

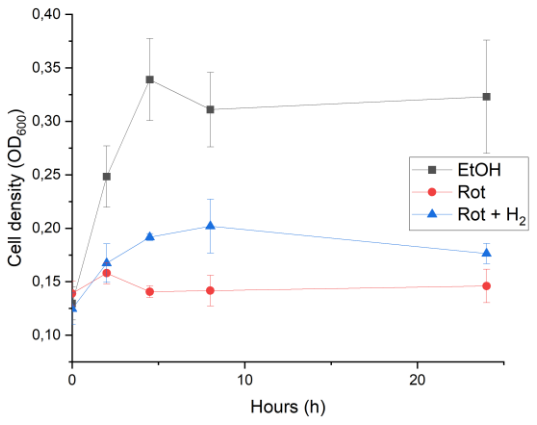

(B)

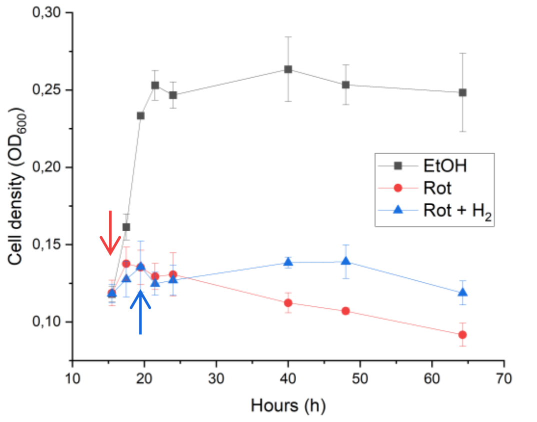

Figure S5. Growth curve of *D. hafniense* strain DCB-2 in Lactate/Fumarate condition with supplement of H_2, (A) prior to inoculation; (B) after 20 h of cultivation. For each condition, triplicates of three different cultures were performed with supplementation of ethanol (control, +EtOH), of rotenone (+Rot) and hydrogen (Rot + H_2). Arrows in panel B indicate the time points at which rotenone (red arrow) and H_2 (blue arrow) were spiked.

References

Jumper, J., Evans, R., Pritzel, A., Green, T., Figurnov, M., Ronneberger, O., et al. (2021). Highly accurate protein structure prediction with AlphaFold. *Nature* 596, 583–589. doi: 10.1038/s41586-021-03819-2

Larkin, M. A., Blackshields, G., Brown, N. P., Chenna, R., McGettigan, P. A., McWilliam, H., et al. (2007). Clustal W and Clustal X version 2.0. *Bioinformatics* 23, 2947–2948. doi: 10.1093/bioinformatics/btm404

Young, T., Niks, D., Hakopian, S., Tam, T. K., Yu, X., Hille, R., et al. (2020). Crystallographic and kinetic analyses of the FdsBG subcomplex of the cytosolic formate dehydrogenase FdsABG from *Cupriavidus necator*. *Journal of Biological Chemistry* 295, 6570–6585. doi: 10.1074/jbc.RA120.013264
